# Effectiveness of electroacupuncture (EA) for the treatment of urinary incontinence (UI) in patients with spinal cord injury (SCI): A protocol of systematic review of randomized controlled trials

**DOI:** 10.1097/MD.0000000000021077

**Published:** 2020-07-24

**Authors:** Tian-Shu Wang, Zeng-Mian Wang, Yu Zhao, Zhao-Chen Tang, Wei-Dong Song, Guan-Kai Wang

**Affiliations:** a Second Ward of Orthopedics Department; b Third Ward of Neurology Department, First Affiliated Hospital of Jiamusi University, Jiamusi, China; c Department of Orthopedics, Huludao Central Hospital, Huludao; d School of Clinical Medicine, Jiamusi University, Jiamusi; e Department of Orthopedics, Second Affiliated Hospital of Mudanjiang Medical University, Mudanjiang; f Department of Orthopedics, Graduate School of Jiamusi University, China.

**Keywords:** effectiveness, electroacupuncture, spinal cord injury, urinary incontinence

## Abstract

**Background:**

The objective of this study is to examine the effectiveness and safety of electroacupuncture (EA) in the treatment of urinary incontinence (UI) in patients with spinal cord injury (SCI).

**Methods:**

All potential studies will be retrieved from the electronic databases of MEDLINE, EMBASE, Cochrane Library, PsycINFO, Web of Science, CBM, and China National Knowledge Infrastructure from origin of each database up to January 31, 2020. Additionally, we will check other resources, such as Google scholar, dissertations, conference proceedings, and reference lists of included studies. No language and publication date limitations will be considered in the literature resources search. All randomized controlled trials using EA for the treatment of UI in patients with SCI will be included. Two independent investigators will perform study selection, data extraction and study quality assessment. If any conflicts occur, we will invite a third investigator to solve them. Cochrane risk of bias will be used for study quality assessment, and RevMan 5.3 software will be employed for statistical analysis.

**Results:**

This study will summarize the most recent evidence to assess the effectiveness and safety of EA for the treatment of UI in patients with SCI.

**Conclusion:**

The results of this study will provide helpful evidence to determine whether EA is effective and safety for the treatment of UI in patients with SCI or not.

**PROSPERO registration number:**

PROSPERO CRD42020165562.

## 1 Introduction

Spinal cord injury (SCI) is a serious and debilitating central nervous system neurological disorder.^[1–3]^ It is reported that about 250,000 and 500,000 new cases annually and most of them are traumatic, with male-to-female of 2:1.^[4,5]^ Several factors result in SCI, such as traffic accidents, violence, sports, and falls.^[6–11]^ Patients with SCI often experience a variety of complications, including pain, spasticity, pressure ulcers, respiratory, cardiovascular, and urinary and bowel disorders, especially urinary incontinence (UI), which leads to very poor quality of life.^[12–16]^

Electroacupuncture (EA) has been widely used to treat numerous diseases around the world.^[17–22]^ Studies suggested that it can effectively manage UI in patients with SCI.^[23–30]^ However, no systematic review has been conducted on this subject. Thus, this study aims to supply sufficient evidence for the clinical application of EA for the treatment of UI following SCI.

## 2 Methods

### 2.1 Study registration

This study was funded and registered on PROSPERO (CRD42020165562). It is reported according to the guidelines of the preferred reporting items for systematic reviews and meta-analysis protocol statement.^[31,32]^

### 2.2 Criteria for including studies

#### 2.2.1 Types of studies

This study will only consider randomized controlled trials (RCTs) assessing the effectiveness and safety of EA for the treatment of UI in patients with SCI for inclusion. We will not limit their language and publication date to all included RCTs.

#### 2.2.2 Types of interventions

The intervention of the trial group only used EA for the treatment of UI in patients with SCI.

The intervention of the control group could use any treatments, such as conventional therapy, medication, and any others. However, we will exclude EA or EA combined with other therapies as comparators.

#### 2.2.3 Types of patients

Regardless of ethnicity, age, sex, educational background, any SCI patients who were diagnosed as UI will be included in this study.

#### 2.2.4 Types of outcome measurements

The primary outcome is the change from baseline in the amount of urine leakage, as measured by the pad-weighing test or other tests.

The secondary outcomes are urination diary, bladder capacity, severity of UI, a 72-hour incontinence episode frequency, clinical symptom scores, the number of participants healed completely within study period and adverse events.

### 2.3 Data sources and search

We will comprehensively search the electronic databases of MEDLINE, EMBASE, Cochrane Library, PsycINFO, Web of Science, CBM, and China National Knowledge Infrastructure from origin of each database up to January 31, 2020. All literature resources will be searched regardless language and publication date. The detailed search strategy of MEDLINE is built (Table 1). Similar search strategies of other electronic databases will be modified.

**Table 1:**
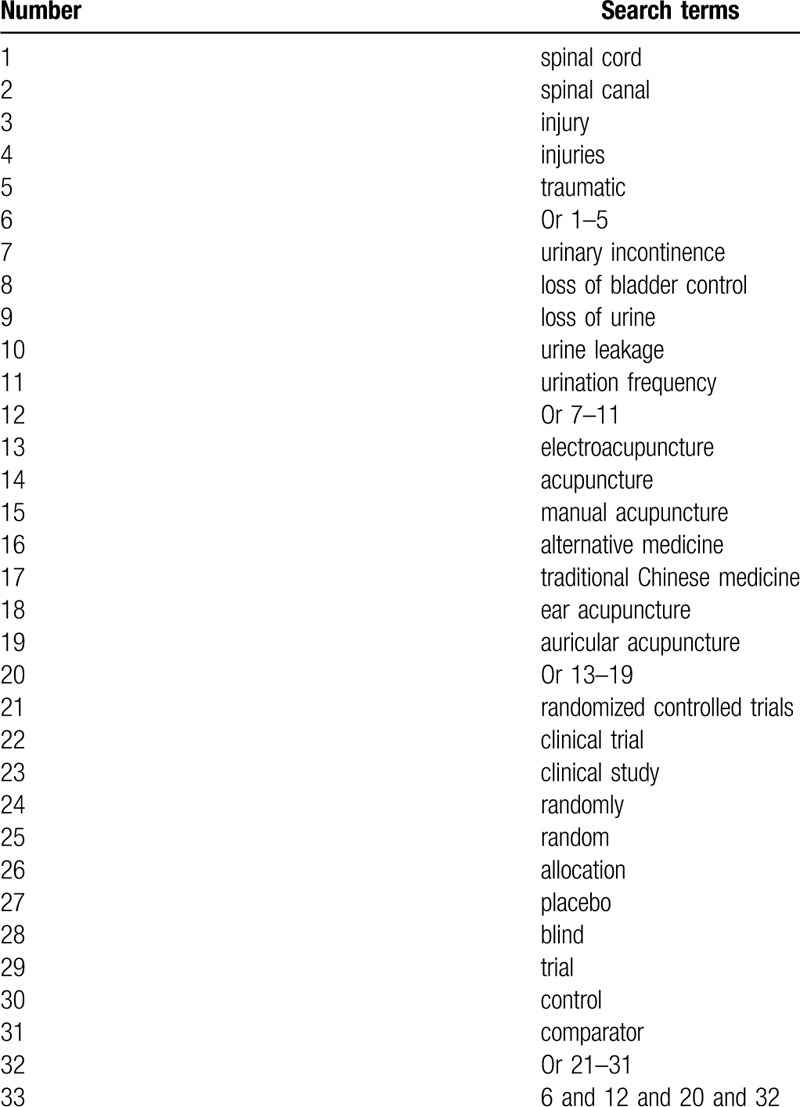
Search strategy for MEDLINE.

In addition, we will search other sources, such as Google scholar, dissertations, conference proceedings, and reference lists of eligible trials.

### 2.4 Data collection and analysis

#### 2.4.1 Study selection

All retrieved literatures will be imported into Endnote X7 and all duplicates will be removed. Two investigators will independently screen the titles and abstracts of all searched literatures, and any unconnected studies will be excluded. Then, full-texts of the remaining studies will be obtained and read cautiously against all inclusion criteria. Any uncertainty between two investigators will be resolved through consulting a third investigator. The procedure of study selection will be exerted in a flow diagram.

#### 2.4.2 Data extraction

Two investigators will independently extract all essential data from each included trial using a predefined data extraction form. Any divergences will be figured out with the help of a third investigator through discussion. We will extract the following information:

Study information: first author, publication year, country, et al.

Patient information: gender, age, race, diagnostic criteria, inclusion and exclusion criteria, et al.

Trial methods: details of randomization, concealment, blind, et al.

Specifics of intervention and controls: treatment duration, dosage, frequency, et al.

Outcome details: primary and secondary outcomes, adverse events, et al.

#### 2.4.3 Missing data dealing with

If there is unclear or insufficient data, we will contact primary authors to request it. If we cannot receive those data, we will only analyze available data. If necessary, we will discuss its potential effects on the study findings.

#### 2.4.4 Study quality assessment

Two investigators will independently appraise study quality using Cochrane risk of bias tool through 7 fields. Each item is graded as high, unclear or low risk of bias. Any differences will be solved by a third investigator through discussion and a consensus will be reached after discussion.

#### 2.4.5 Subgroup analysis

We will carry out subgroup analysis to identify the possible factors that may result in significant heterogeneity based on the different interventions, controls and outcome indicators.

#### 2.4.6 Sensitivity analysis

We will perform sensitivity analysis to test the robustness and stability of study findings by excluding low quality trials.

#### 2.4.7 Reporting bias

We will examine the reporting bias using funnel plot and Egger regression test if more than 10 included trials are included.^[33,34]^

### 2.5 Data synthesis

RevMan 5.3 software will be used to undertake statistical analysis. To assess the extracted data, mean difference or standardized mean difference and 95% confidence intervals will be used for continuous data. For dichotomous data, we will use risk ratio and 95% confidence intervals. Statistical heterogeneity across eligible trials will be inspected using *I*^*2*^ statistics. *I*^*2*^ ≤ 50% means fair heterogeneity, and a fixed-effect model will be examined. If sufficient outcome data is extracted, we will conduct a meta-analysis. *I*^*2*^ > 50% suggests apparent heterogeneity, and a random-effect model will be used. We will carry out subgroup analysis to investigate the sources of heterogeneity. If the sources of heterogeneity cannot be identified, synthetic analysis will not be performed and descriptive analysis will be adopted.

#### 2.5.1 Quality of evidence

The quality of evidence for major outcomes will be appraised by 2 independent investigators using grading of recommendations assessment, development and evaluation.^[35]^ Any disagreements will be disentangled by another investigator through consultation.

### 2.6 Dissemination and ethics

We expect to publish this study on a peer-reviewed journal. This study will not inquire ethic approval because it will not collect individual patient data.

## 3 Discussion

UI is a progressive disorder in patients with SCI.^[23–30]^ EA is currently used in the treatment of UI after SCI, relieving clinical symptoms, frequency and severity of UI. Although previous clinical studies have reported that EA has positive therapeutic effects on UI following SCI, all conclusions drawn are based on the individual trial. Thus, this study is designed to systematically and comprehensively assess the effectiveness and safety of EA for the treatment of UI in patients with SCI. The results of this study will provide evidence to determine whether EA is an effective and safety treatment for UI following SCI, which may benefit clinical practice and patients.

### 3.1 Uncited references

^[17,18]^.

## Author contributions

**Conceptualization:** Tian-Shu Wang, Wei-Dong Song, Zhao-Chen Tang, Yu Zhao, Guan-kai Wang.

**Data curation:** Zeng-Mian Wang, Yu Zhao.

**Formal analysis:** Tian-Shu Wang, Zeng-Mian Wang, Wei-Dong Song, Zhao-Chen Tang.

**Investigation:** Yu Zhao.

**Methodology:** Tian-Shu Wang, Zeng-Mian Wang, Wei-Dong Song, Zhao-Chen Tang, Guan-Kai Wang.

**Project administration:** Yu Zhao.

**Resources:** Tian-Shu Wang, Zeng-Mian Wang, Wei-Dong Song, Zhao-Chen Tang, Guan-Kai Wang.

**Software:** Tian-Shu Wang, Zeng-Mian Wang, Wei-Dong Song, Zhao-Chen Tang, Guan-Kai Wang.

**Supervision:** Yu Zhao.

**Validation:** Tian-Shu Wang, Zeng-Mian Wang, Zhao-Chen Tang, Yu Zhao, Guan-Kai Wang.

**Visualization:** Tian-shu Wang, Wei-dong Song, Zhao-chen Tang, Yu Zhao.

**Writing – original draft:** Tian-Shu Wang, Zeng-Mian Wang, Wei-Dong Song, Zhao-Chen Tang, Yu Zhao, Guan-Kai Wang.

**Writing – review & editing:** Tian-Shu Wang, Zeng-Mian Wang, Wei-Dong Song, Yu Zhao, Guan-Kai Wang.

## References

[1] Ning GZ Wu Q Li YL Epidemiology of traumatic spinal cord injury in Asia: a systematic review. J Spinal Cord Med 2012;35:229–39. 22925749 10.1179/2045772312Y.0000000021 PMC3425879

[2] Lu Y Yang J Wang X Research progress in use of traditional Chinese medicine for treatment of spinal cord injury. Biomed Pharmacother 2020;127:110136. 32335299 10.1016/j.biopha.2020.110136

[3] Rabinstein AA Traumatic spinal cord injury. Continuum (Minneap Minn) 2018;24:551–66. 29613899 10.1212/CON.0000000000000581

[4] Hachem LD Ahuja CS Fehlings MG Assessment and management of acute spinal cord injury: From point of injury to rehabilitation. J Spinal Cord Med 2017;40:665–75. 28571527 10.1080/10790268.2017.1329076 PMC5778930

[5] WHO-Spinal Cord Injury. WHO, Fact sheet N°384 (2013). Available online at: https://www.who.int/news-room/fact-sheets/detail/spinal-cord-injury [accessed date March 1, 2020].

[6] Eckert MJ Martin MJ Trauma: spinal cord injury. Surg Clin North Am 2017;97:1031–45. 28958356 10.1016/j.suc.2017.06.008

[7] Suriá Martínez R Factors associated with empowerment in people with a spinal cord injury due to traffic accidents. Gac Sanit 2015;29: Suppl 1: 49–54. 26342418 10.1016/j.gaceta.2014.11.011

[8] Moslavac S Dzidić I Kejla Z Neurological outcome in road traffic accidents with spinal cord injury. Coll Antropol 2008;32:583–6. 18756914

[9] Kroll T Rehabilitative needs of individuals with spinal cord injury resulting from gun violence: the perspective of nursing and rehabilitation professionals. Appl Nurs Res 2008;21:45–9. 18226763 10.1016/j.apnr.2006.06.001

[10] Franz M Richner L Wirz M Physical therapy is targeted and adjusted over time for the rehabilitation of locomotor function in acute spinal cord injury interventions in physical and sports therapy. Spinal Cord 2018;56:158–67. 29057989 10.1038/s41393-017-0007-5

[11] Wilson A Kurban D Noonan VK Falls during inpatient rehabilitation in spinal cord injury, acquired brain injury, and neurologmusculoskeletal disease programs. Spinal Cord 2020;58:334–40. 31641202 10.1038/s41393-019-0368-z

[12] Qi Z Middleton JW Malcolm A Bowel dysfunction in spinal cord injury. Curr Gastroenterol Rep 2018;20:47. 30159690 10.1007/s11894-018-0655-4

[13] Dinh A Bouchand F Davido B Management of established pressure ulcer infections in spinal cord injury patients. Med Mal Infect 2019;49:9–16. 29937316 10.1016/j.medmal.2018.05.004

[14] Shiao R Lee-Kubli CA Neuropathic pain after spinal cord injury: challenges and research perspectives. Neurotherapeutics 2018;15:635–53. 29736857 10.1007/s13311-018-0633-4 PMC6095789

[15] Finnerup NB Neuropathic pain and spasticity: intricate consequences of spinal cord injury. Spinal Cord 2017;55:1046–50. 28695904 10.1038/sc.2017.70

[16] Sezer N Akkuş S Uğurlu FG Chronic complications of spinal cord injury. World J Orthop 2015;6:24–33. 25621208 10.5312/wjo.v6.i1.24 PMC4303787

[17] Wu SY Lin CH Chang NJ Combined effect of laser acupuncture and electroacupuncture in knee osteoarthritis patients: A protocol for a randomized controlled trial. Medicine (Baltimore) 2020;99:e19541. 32195960 10.1097/MD.0000000000019541 PMC7220484

[18] Wang Z Dong H Wang Q Effects of electroacupuncture on anxiety and depression in unmarried patients with polycystic ovarian syndrome: secondary analysis of a pilot randomised controlled trial. Acupunct Med 2019;37:40–6. 30843421 10.1136/acupmed-2017-011615

[19] Ning Y Jia H Chen P Efficacy and safety of electroacupuncture on metabolic syndrome due to olanzapine and risperidone: Study protocol for a randomized controlled pilot trial. Medicine (Baltimore) 2019;98:e17237. 31567988 10.1097/MD.0000000000017237 PMC6756590

[20] Liu T Lu Y Yu J Effect of auricular electroacupuncture combined with body acupuncture in improving the consciousness of patients after traumatic brain injury: Study protocol for a randomized controlled trial. Medicine (Baltimore) 2019;98:e16587. 31348298 10.1097/MD.0000000000016587 PMC6709251

[21] Zhong S Huang H Xie J Application of electroacupuncture for postoperative pain management after total knee arthroplasty: a study protocol for a single-blinded, randomised placebo-controlled trial. BMJ Open 2019;9:e026084. 10.1136/bmjopen-2018-026084 PMC6500353 30962235

[22] Wang W Liu Y Zhao J Electroacupuncture versus manual acupuncture in the treatment of plantar heel pain syndrome: study protocol for an upcoming randomised controlled trial. BMJ Open 2019;9:e026147. 10.1136/bmjopen-2018-026147 PMC6500181 30948595

[23] Xian DL Wang XB Lin R Fuyuan Huoxue Decoction combined with electroacupuncture for neurological function rehabilitation after spinal cord injury. Chin J Exp Pharmacol 2020;20:1–0.

[24] Cheng XK Sun YC Clinical study on electroacupuncture for treatment of neurogenic bladder incontinence of spinal cord injury. Shanghai J Acupunct Moxibust 2019;38:646–9.

[25] Zhu N Ma Q Zhang J Clinical observation of acupuncture combined with pelvic-sacral muscle stimulation on neurogenic bladder with spinal cord injury. Ningxia Med J 2017;39:553–5.

[26] Lu CJ Li X Zhang HR Observation on the therapeutic effect of different electroacupuncture waveforms on neurogenic bladder with spinal cord injury. Shanghai J Acupunct Moxibust 2016;35:1442–4.

[27] Meng ZX Wang T Yin ZL Clinical study on electroacupuncture combined with transperineal BTX-A injection for neurogenic bladder after spinal cord injury. Chin Acupunc 2015;35:17–20. 25906559

[28] Feng XJ Wei XC Wu JX Treatment of 23 cases of neurogenic bladder with incomplete spinal cord injury by electroacupuncture. J Anhui Univ Trad Chin Med 2014;33:43–6.

[29] Ma L Chen XY Clinical observation of electroacupuncture at Ba li points combined with warm moxibustion for the treatment of 30 cases of spinal cord-induced urinary incontinence. Hunan J Trad Chin Med 2012;28:81–3.

[30] Cong HL Liao LM Si T The effect of electroacupuncture regulating sacral 3 nerve on neurogenic detrusor overactivity. Chin J Urol 2010;11:741–4.

[31] Shamseer L Moher D Clarke M PRISMA-P Group. Preferred reporting items for systematic review and meta-analysis protocols (PRISMA-P) 2015 elaboration and explanation. BMJ 2015;349:g7647. 10.1136/bmj.g7647 25555855

[32] Moher D Shamseer L Clarke M Preferred reporting items for systematic review and meta-analysis protocols (PRISMA-P) 2015 statement. Syst Rev 2015;4:1. 25554246 10.1186/2046-4053-4-1 PMC4320440

[33] Sutton AJ Duval SJ Tweedie RL Empirical assessment of effect of publication bias on meta-analyses. BMJ 2000;320:1574–7. 10845965 10.1136/bmj.320.7249.1574 PMC27401

[34] Egger M Davey Smith G Schneider M Bias in meta-analysis detected by a simple, graphical test. BMJ 1997;315:629–34. 9310563 10.1136/bmj.315.7109.629 PMC2127453

[35] Guyatt GH Oxman AD Vist GE GRADE: an emerging consensus on rating quality of evidence and strength of recommendations. BMJ 2008;336:924–6. 18436948 10.1136/bmj.39489.470347.AD PMC2335261

